# Intrathecal dexmedetomidine as an adjuvant to plain ropivacaine for spinal anesthesia during cesarean section: a prospective, double-blinded, randomized trial for ED_50_ determination using an up-down sequential allocation method

**DOI:** 10.1186/s12871-023-02275-x

**Published:** 2023-09-25

**Authors:** Xiaofei Mo, Fa Huang, Xiaoying Wu, Jumian Feng, Jiequn Zeng, Jinghui Chen

**Affiliations:** Department of Anesthesiology, Guangzhou Women and Children’s Medical Center, Guangzhou Medical University, Guangzhou, China

**Keywords:** Cesarean section, Dexmedetomidine, Ropivacaine, Spinal anesthesia

## Abstract

**Background:**

Intrathecal dexmedetomidine, as an adjuvant to local anesthetics, has been reported to improve the quality of spinal anesthesia and reduce the required local anesthetic dose. However, the optimal dosage regimen for intrathecal dexmedetomidine combined with plain ropivacaine for cesarean section (CS) remains undetermined. The present study aimed to determine the median effective dose (ED_50_) of intrathecal dexmedetomidine as an adjuvant to plain ropivacaine for spinal anesthesia during CS.

**Methods:**

Sixty parturients undergoing CS were randomly assigned to either group: plain ropivacaine 8 mg (Group Rop_8_) or plain ropivacaine 10 mg (Group Rop_10_). The initial dosage of intrathecal dexmedetomidine in each group was 5 µg. The effective dose was defined as a bilateral sensory block at the level of T6 or above to pinprick attained within 10 min after intrathecal injection, without the need for supplementary intraoperative epidural anesthesia. Effective or ineffective responses were determined, followed by a 1 µg increment or decrement in the dose of intrathecal dexmedetomidine for the next parturient using up-down sequential allocation. ED_50_ were calculated using probit regression.

**Results:**

The ED_50_ of intrathecal dexmedetomidine with plain ropivacaine was 5.9 µg (95% confidence interval [CI], 4.9–7.4 µg) in Group Rop_8_ and 3.1 µg (95% CI, 0.1–4.8 µg) in Group Rop_10_ (*P* < 0.05). Hemodynamic stability, side effects, patient satisfaction and neonatal outcomes were comparable between the two groups.

**Conclusions:**

The present data suggested that the ED_50_ of intrathecal dexmedetomidine as an adjuvant to 8 mg and 10 mg plain ropivacaine in spinal anesthesia during cesarean section was approximately 6 µg and 3 µg, respectively.

**Trial registration:**

Chinese Clinical Trial Registry, identifier: ChiCTR2200055928.

**Supplementary Information:**

The online version contains supplementary material available at 10.1186/s12871-023-02275-x.

## Background

Regional anesthesia (spinal or epidural anesthesia) has been recommended as the preferred option for elective cesarean section compared to general anesthesia. The main reason for the preference is the negligible maternal mortality associated with gastric acid aspiration of mothers. Spinal anesthesia for cesarean section is thought to be advantageous due to its rapid onset of nerve blockade and reliable performance [1]. Improving the quality of anesthesia can improve maternal and infant outcomes of patients with cesarean section. Studies [2, 3] have shown that reducing the intrathecal dose of local anesthetics can reduce the incidence and severity of hypotension, decrease nausea and vomiting, aid early ambulation owing to faster recovery from motor block, and improve overall maternal satisfaction. However, a lower dose of spinal anesthetic is associated with an increased risk of spinal anesthesia failure, intraoperative pain, and a shorter duration of effective anesthesia with a slower onset [3, 4]. Opioids are the most commonly used local anesthetic adjuvants to improve the quality of anesthesia [2, 3, 5]. Unfortunately, intrathecal opioids may increase the occurrence of nausea and vomiting, urinary retention, pruritus or even respiratory depression.

Dexmedetomidine has been increasingly used as a local anesthetic adjuvant for spinal anesthesia. The combination was associated with a long list of benefits, including reduced use of analgesics, improved intraoperative nerve blockade, shortened onset time of the sensory or motor block, lowered occurrence of shivering, prolonged postoperative analgesia, and reduced postoperative pain score in cesarean Sects. [6–13]. The doses of intrathecal dexmedetomidine used in clinical studies of cesarean section have ranged from 2.5 to 10 µg [7–14]. However, the optimal dose of intrathecal dexmedetomidine with plain ropivacaine has not been determined.

Therefore, our primary aim was to determine the median effective dose (ED_50_) of intrathecal dexmedetomidine coadministered with different doses of plain ropivacaine in parturients undergoing cesarean section using an up-down sequential allocation methodology.

## Methods

### Study design and participants

Sixty parturients scheduled for elective cesarean section were enrolled in the present study. Patient inclusion criteria were ASA Physical Status II, aged 18 to 45 years, term gestation (≥ 37 weeks), singleton pregnancy, and scheduled for elective cesarean section under combined spinal-epidural anesthesia (CSEA). Exclusion criteria were contraindications to neuraxial anesthesia, BMI ≥ 40 kg·m^− 2^, severe pregnancy complications (e.g., hemorrhage, preeclampsia, heart failure, severe anemia and diabetes mellitus with complications), use of sedative or analgesic drugs 2 h before surgery, preoperative heart rate (HR) < 50 beats·min^− 1^ with cardiac conduction or rhythm abnormalities, allergy to drugs used in the study, or known fetal abnormalities.

### Ethics

The study protocol was approved by the local ethics committee, the Guangzhou Women and Children’s Medical Centre Ethics Committee, Guangzhou, China, on March 11, 2022 (reference number 2021-232B00, Chairperson Professor Sitang Gong). The protocol was registered at the Chinese Clinical Trial Registry (Registration number: ChiCTR2200055928, Date of registration: 26/01/2022) before the start of enrollment on 14/03/2022. This study complied with the Consolidated Standards of Reporting Trials (CONSORT) guidelines. All participants provided written informed consent prior to study commencement.

### Randomization and blinding

Consenting parturients were randomly allocated into Group Rop_8_ (intrathecal ropivacaine 8 mg + dexmedetomidine) or Group Rop_10_ (intrathecal ropivacaine 10 mg + dexmedetomidine) using a computer-generated randomization list. The entire randomization sequence was generated prior to enrollment of the first participant. Allocation was blinded by sequentially numbered sealed opaque envelopes that were opened at the time of randomization. A staff member who did not participate in the study organized and kept the randomization code until study completion. Patients, anesthesiologists, obstetricians and researchers were blinded to the group assignments.

### Anesthetic procedure

All patients fasted for 6 h and discontinued fluid intake 2 h before cesarean section. No patient was given premedication. After an intravenous catheter was placed, the patient was transported to the operating room, where standard ASA monitors were placed and a volume of 500 mL of Ringer’s lactate solution was started. With the patient in a left lateral decubitus position, a 25-gauge pencil-point needle was introduced into the subarachnoid space at the L3-L4 or L2-L3 interspace using the single-space needle-through-needle technique in a standard sterile fashion. After the return of clear cerebrospinal fluid, a 2.5 mL freshly prepared mixed solution containing plain ropivacaine (Naropin®, AstraZeneca AB Company, Sodertalje, Sweden) 8 mg + dexmedetomidine (Dexmedetomidine Hydrochloride Injection, 200 µg per 2 mL, Jiangsu Yangzi River Pharmaceutical Co., Ltd.; preservative-free and contains no additives or chemical stabilizers) (Group Rop_8_) or plain ropivacaine 10 mg + dexmedetomidine (Group Rop_10_), diluted with preservative-free normal saline to achieve the desired volume, was injected over a duration of approximately 10 s. Then, an epidural catheter was placed 4 cm into the epidural space, and no local anesthetic test dose was injected at this time. The parturient was then immediately turned in the supine position with a 15° left tilt. All subjects received a prophylactic intravenous phenylephrine infusion at 0.5 µg·kg^− 1^·min^− 1^ initiated at the time of spinal injection.

The mixed solutions for spinal anesthesia were previously prepared by an independent anesthesiologist not involved further in the trial. A second anesthesiologist who was blinded to the details of the mixed solutions performed the CSEA procedure and intraoperative management as well as the subsequent assessments. A 1 mL insulin syringe was used for measuring volumes ≤ 1 mL. Dexmedetomidine for the present study was prepared by withdrawing 0.1 mL (10 µg) of dexmedetomidine from an ampoule of 100 µg·mL^− 1^ dexmedetomidine into an insulin syringe containing 10 divisions. This 10 µg of dexmedetomidine was then further diluted with preservative-free normal saline to make up a total volume of 1 mL, i.e., 1 µg·division^− 1^.

The dose of dexmedetomidine for the first patient in each group was 5 µg. The dose of dexmedetomidine for the following patient was determined by the response of the previous patient to the intrathecal mixed solution in the same group according to the up-down sequential allocation method. If the response of the previous patient was effective, the dose of intrathecal dexmedetomidine for the subsequent patient was decreased by 1 µg. If the response of the patient was ineffective, the dose for the subsequent patient was increased by 1 µg. In case of an effective response in 1 µg or an ineffective response in 10 µg, the dose for the subsequent patients would remain the same until an ineffective response or an effective response prompted the anesthesiologist to increase or decrease the dose, respectively. An effective intrathecal block was defined as a bilateral sensory level to pinprick of T6 or above, which was achieved within 10 min after injection of spinal solution without the requirement of additional intraoperative epidural anesthetic. The ineffective intrathecal block was defined as failure to obtain a bilateral sensory level to pinprick of T6 within 10 min of intrathecal drug administration, or additional analgesia was required to complete surgery because of either a visual analog score (VAS; 0–10; 0 = no pain and 10 = worse pain imaginable) greater than 2, or the patient’s request for additional analgesia, despite achieving T6 sensory level block. In cases of ineffective response, supplemental epidural anesthesia consisting of 2% lidocaine was administered as 5 mL bolus injections, repeated every 5 min if necessary [8]. The sensory block level was assessed bilaterally at the midclavicular line with a pinprick test every 2 min for the first 10 min after spinal injection, followed by tests at 10-min intervals until the end of surgery. No intrathecal opioid was added to the spinal cord for all patients. At the end of the surgery, the same solution at a single-dose of ropivacaine (1 mg) and hydromorphone (0.6 mg) diluted with saline to 8 mL was administered to both groups via the epidural catheter. Patient-controlled intravenous analgesia (PCIA) was then immediately administered with an electronic analgesia pump filled with sufentanil (100 µg) and flurbiprofen (200 mg) diluted with saline to 100 mL. The PCIA pump was set as follows: 2 mL/h continuous dose, 2 mL/h self-controlled dose, and 10 min lock time.

### Measurements

The primary outcome was effective intrathecal block. Secondary outcomes included: the onset time of sensory block, which was defined as the time taken from intrathecal injection to T10 dermatome sensory block level being achieved; the highest level of sensory block within 10 min after intrathecal injection and the time taken to reach this maximal sensory block; the duration of sensory block, which was defined as the time to 2 segment regression checked every 10 min after achieving peak sensory block level; the onset time of motor block (assessed in both lower extremities using the Bromage scale [15]; 0 = no motor paralysis, 1 = Unable to raise the extended leg, but able to move knee and foot, 2 = Unable to raise the extended leg as well as flex knees, able to move foot, and 3 = Unable to flex the ankle, foot or knee), which was defined as the time from intrathecal injection to Bromage Score = 1; the duration of motor block, which was defined as the time between completion of intrathecal injection to return of Bromage score = 0; VAS scores at skin incision, fetal delivery, peritoneal closure and skin closure; the consumption of epidural lidocaine; the hemodynamic parameters of parturient including blood pressure (BP) & HR, which were checked at baseline, at 2-min intervals for the first 10 min after spinal injection, and at 10-min intervals until the end of surgery; sedation, visceral traction response, abdominal muscle relaxation, and shivering scores, which were graded according to Supplemental table; the parturient’s satisfaction, which was evaluated with a 5-point scale (1 = very disappointed; 2 = disappointed; 3 = so-so; 4 = satisfactory; 5 = very pleased) at the end of surgery; the blood gas analysis of the umbilical vein immediately after delivery; Apgar scores, which were assessed at 1 and 5 min by a pediatrician who was unaware of the treatment group.

Hypotension (defined as systolic BP 20% less than the baseline value or less than 90 mmHg) was treated with 40 µg intravenous phenylephrine and was repeated as needed. Bradycardia, defined as HR < 50 beats·min^− 1^, was treated with 0.5 mg of atropine intravenously. Baseline BP and HR were calculated as the average of three readings at admission. Hypoxemia, defined as oxygen saturation below 95%, was treated with mask oxygen inhalation.

### Statistical analysis

Due to the nonindependence and unknown distribution of data associated with an up-down sequential allocation method, the exact sample size needed for a prespecified precision of the estimation of ED_50_ could not be determined in advance. According to the stop rule of the updown sequential method, simulation studies suggest that enrolling at least 20–40 patients will provide a stable estimate of ED_50_ for most cases [16, 17]. Therefore, in this study, we decided to enroll 30 patients for each group.

Statistical analyses were performed using SPSS (version 25.0, IBM Corp., Armonk, NY, USA). Data were presented as the mean ± standard deviation (SD), the median (interquartile range, IQR), or the number (proportion) as appropriate. Values for ED_50_ (95% confidence interval [CI]) were estimated using probit regression. For the normality test, the Kolmogorov‒Smirnov test was performed. The differences between groups were compared using Student’s *t* test (normally distributed data) or the Mann‒Whitney *U* test (skewed data). Categorical variables and proportions were analyzed using the Pearson chi-square test or Fisher’s exact test as appropriate. Repeated measures of hemodynamic values were analyzed by a two-way repeated-measures analysis of variance (ANOVA) with adjustment of baseline as a covariate. *P* < 0.05 was considered statistically significant.

## Results

**Fig. 1:**
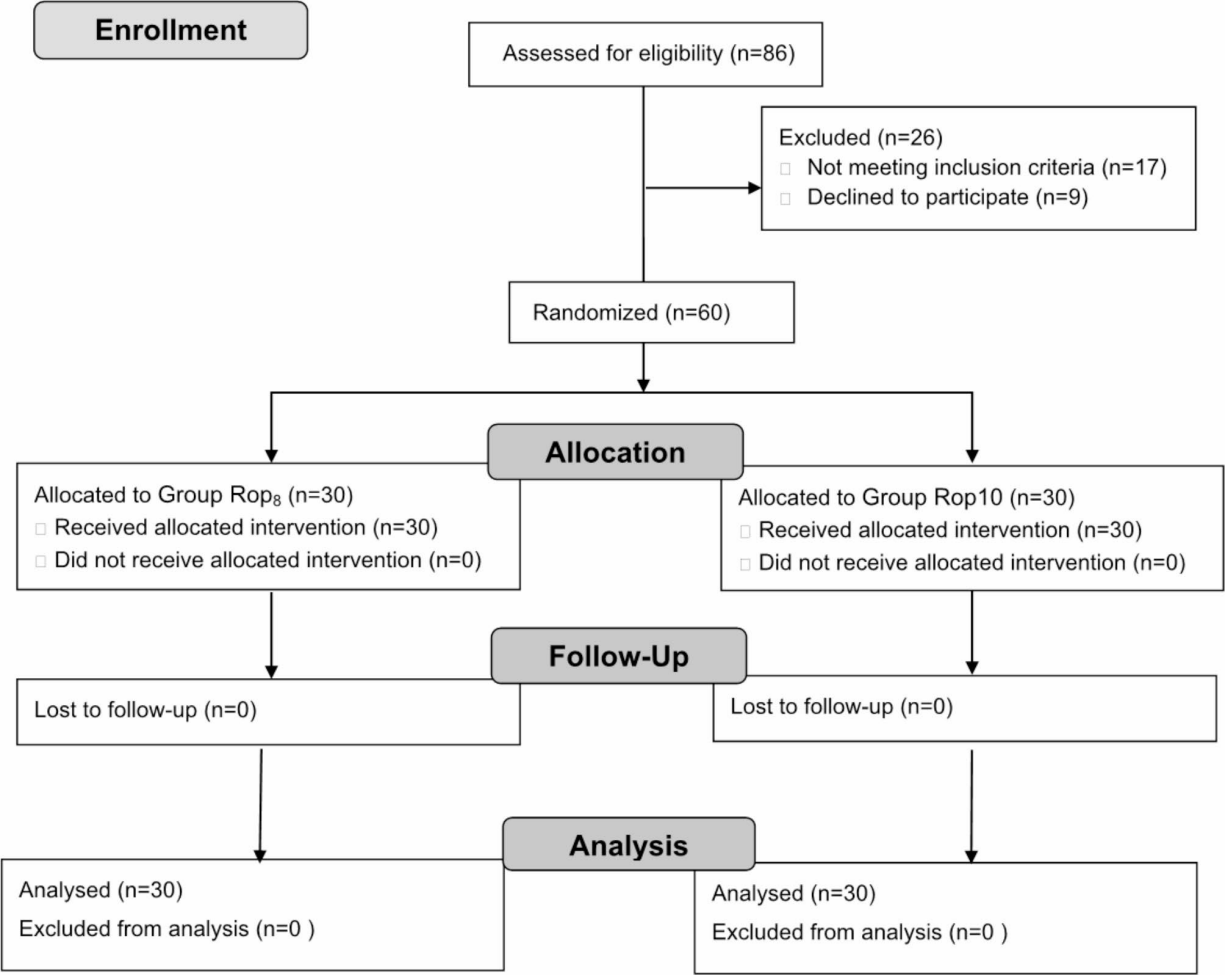
Flowchart of the study

Eighty-six patients were screened for this study. Nine declined to participate, and 17 did not meet the inclusion criteria. Sixty patients (n = 30 patients per group) were enrolled and randomly assigned to a dose group. They all completed the study according to the protocol and were included in the analysis (Fig. 1). Patient demographics are shown in Table 1.

**Table 1:** Patient Characteristics

Parameters	Group Rop_8_ (n = 30)	Group Rop_10_ (n = 30)
Maternal age (years)	34 ± 4	33 ± 3
Weight (kg)	68 ± 8	69 ± 8
Height (cm)	159 ± 4	161 ± 5
Body mass index (kg/m^2^)	27 ± 3	27 ± 2
Gestation (weeks)	39 (38 to 39)	39 (38 to 39)
Duration of surgery (min)	40 ± 9	43 ± 11

**Note**: Data are presented as mean ± SD or median (interquartile range)

**Fig. 2:**
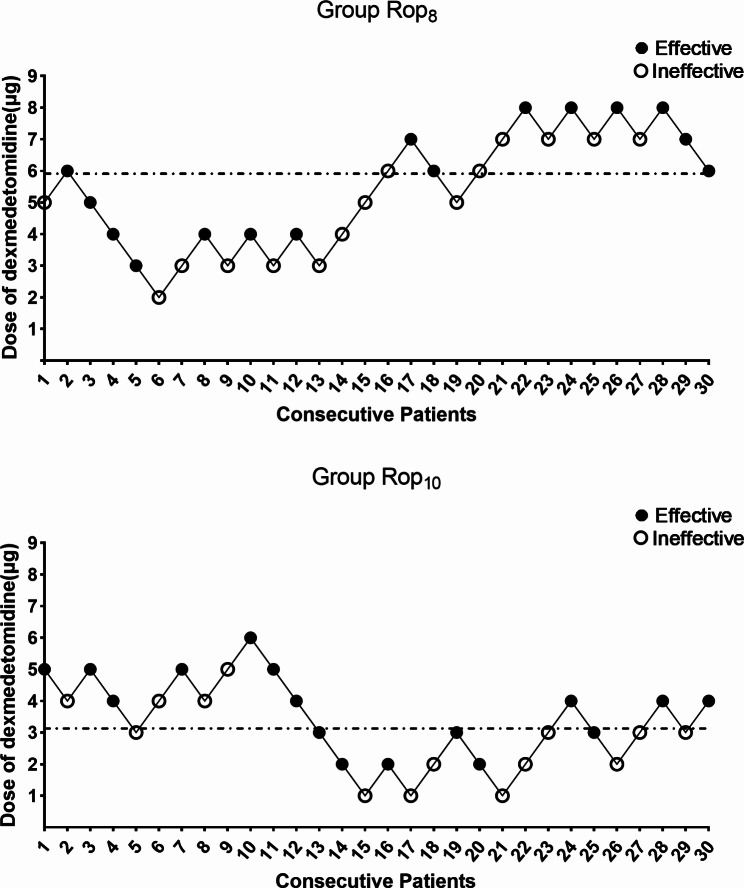
Individual response to intrathecal dexmedetomidine coadministered with ropivacaine at the corresponding dose (µg). The ED_50_ of intrathecal dexmedetomidine coadministered with ropivacaine for cesarean section was 5.9 µg (95% CI, 4.9 to 7.4 µg) in Group Rop_8_ and 3.1 µg (95% CI, 0.1 to 4.8 µg) in Group Rop_10_ using probit regression. An effective intrathecal block is denoted by a filled circle, while an ineffective block is denoted by an open circle. Dashed lines indicate the position of the estimates of ED_50_

**Fig. 3:**
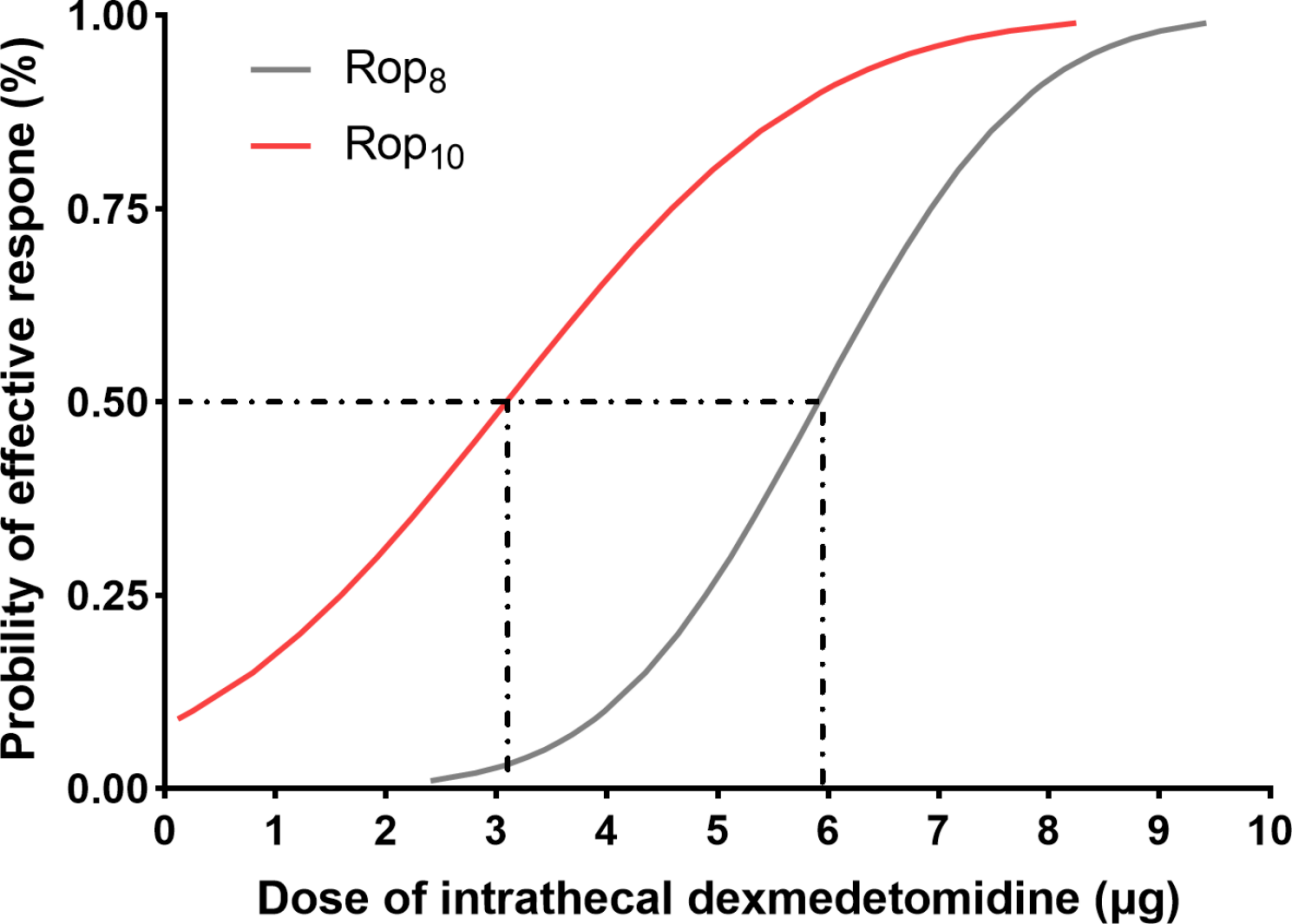
Dose‒response curves for intrathecal dexmedetomidine coadministered with ropivacaine for cesarean section in the two groups derived from probit regression analysis. Dashed lines indicate the position of the estimates of ED_50_

The sequences of cases are illustrated in Fig. 2. The ED_50_ of intrathecal dexmedetomidine combined with plain ropivacaine was 5.9 µg (95% CI, 4.9 to 7.4 µg) in Group Rop_8_ and 3.1 µg (95% CI, 0.1 to 4.8 µg) in Group Rop_10_ (Fig. 2). The ED_50_ value in Group Rop_10_ was significantly lower than that in Group Rop_8_ (*P* < 0.05). Dose‒response curves for intrathecal dexmedetomidine coadministered with plain ropivacaine for cesarean section, derived from probit regression analysis, are shown in Fig. 3.

Characteristics of sensory block and motor block between the two groups or between the subgroups are summarized in Table 2. There was no incidence of unilateral sensory block. The onset time of the sensory block, the time to the highest sensory block level and the duration of the sensory block were similar in both groups (Table 2). The onset time for Bromage scores of 1 & 2 to develop was faster in Group Rop_10_ than in Group Rop_8_, but there was no statistically significant difference in the onset time for Bromage scores to reach 3 (Table 2). The proportion of patients with incomplete motor block was 10.0% (3/30) in Group Rop_8_ and 3.3% (1/30) in Group Rop_10_ during the operation, but the difference was not statistically significant (*P* = 0.612) (Table 2). The duration of motor block was longer in Group Rop_10_ than in Group Rop_8_, but the difference was not statistically significant (*P* = 0.145) (Table 2).

**Table 2:** Characteristics of Sensory Block and Motor Block

Parameters	Group Rop_8_ (n = 30)					Group Rop_10_ (n = 30)						*P*-value
	Dexmedetomidine dosage					Dexmedetomidine dosage						
	4 µg (n = 3)	5 µg (n = 10)	6 µg (n = 10)	7 µg (n = 5)	8 µg (n = 2)	1 µg (n = 3)	2 µg (n = 6)	3 µg (n = 7)	4 µg (n = 8)	5 µg (n = 5)	6 µg (n = 1)	
**Sensory block (to pinprick)**												
Highest sensory block (n, %)												
T2	0	0	0	0	0	0	1 (16.7)	0	1 (12.5)	0	0	-
T3	0	0	1 (10.0)	0	0	0	0	0	1 (12.5)	0	1 (100.0)	-
T4	0	2 (20.0)	3 (30.0)	0	0	0	0	1 (14.3)	2 (25.0)	2 (40.0)	0	-
T5	0	1 (10.0)	2 (20.0)	2 (40.0)	1 (50.0)	0	1 (16.7)	1 (14.3)	0	0	0	-
T6	0	1 (10.0)	0	2 (40.0)	1 (50.0)	0	1 (16.7)	1 (14.3)	2 (25.0)	2 (40.0)	0	-
T7	0	0	0	1 (20.0)	0	0	0	0	0	0	0	-
T8	1 (33.3)	3 (30.0)	0	0	0	2 (66.7)	1 (16.7)	1 (14.3)	0	0	0	-
T9	0	1 (10.0)	0	0	0	0	2 (33.3)	1 (14.3)	1 (12.5)	0	0	-
T10	2 (66.7)	2 (20.0)	4 (40.0)	0	0	1 (33.3)	0	2 (28.6)	1 (12.5)	1 (20.0)	0	-
Onset time to T10 (min)	4.3 ± 2.8					4.3 ± 2.8						1
Time to highest level (min)	9.0 ± 2.1					8.6 ± 2.9						0.543
Duration of sensory block (min)	169.7 + 62.0					192.4 + 72.4						0.197
**Motor block**												
Bromage Score (n, %)												
0	0	0	0	0	0	0	0	0	0	0	0	-
1	0	0	0	0	0	0	0	0	0	0	0	-
2	1 (33.3)	1 (11.1)	1 (11.1)	0	0	0	0	0	1 (12.5)	0	0	-
3	2 (66.7)	9 (88.9)	9 (88.9)	5 (100.0)	2 (100.0)	3 (100.0)	6 (100.0)	7 (100.0)	7 (87.5)	5 (100.0)	1 (100.0)	-
No. of patients with incomplete motor block (n, %)	3 (10.0)					1 (3.3)						0.612
Onset time to Bromage score = 1 (min)	7.37 ± 6.27					3.93 ± 2.12						0.006*
Onset time to Bromage score = 2 (min)	11.03 ± 8.16					7.10 ± 2.99						0.016*
Onset time to Bromage score = 3 (min)	15.78 ± 8.34					14.24 ± 8.13						0.472
Duration of motor block (min)	343.6 ± 73.1					372.5 ± 78.4						0.145

**Notes**: Sensory block refer to the highest level of block within 10 min after intrathecal injection. Motor block refer to the greatest degree of block during the surgery. Data are presented as number (percentage) or mean ± SD. *P < 0.05 versus Group Rop_8_

There was no significant difference in systolic BP, diastolic BP or HR between the two groups by two-way repeated-measures ANOVA. The vital-sign parameters can be seen in the Supplemental figure.

No patient had hypoxemia. The incidence of hypotension, bradycardia, nausea & vomiting, sedation score, visceral traction response score, muscle relaxation score, shivering score, dose of supplemental lidocaine, VAS at different time points at skin incision, fetal delivery, peritoneal closure and satisfaction of patients was comparable between the 2 groups (Table 3).

**Table 3:** Exploratory outcomes

Parameters	Group Rop_8_ (n = 30)	Group Rop_10_ (n = 30)	*P*-value	
Hypoxemia (n, %)	0	0	1	
Bradycardia (n, %)	1 (3.3)	1 (3.3)	1	
Hypotension (n, %)	9 (30.0)	8 (26.7)	0.774	
Nausea and vomiting (n, %)	2 (6.7)	2 (6.7)	1	
Visceral tract reaction 1–2–3 (n, %)	25–5–0	28–2–0	0.228	
Shivering level 0–1–2–3 (n, %)	24–3–3–0	24–2–4–0	0.842	
Muscle relaxation 1–2–3 (n, %)	29–1–0	29–1–0	1	
Sedation level 0–1–2–3 (n, %)	15–4–11–0	21–5–4–0	0.112	
Lidocaine (ml)	6.67 ± 2.44	5.77 ± 1.88	0.291	
VAS of pain				
At skin incision	0	0	1	
At fetal delivery	0.70 ± 1.09	0.37 ± 0.72	0.167	
At peritoneal closure	0.20 ± 0.67	0.23 ± 0.73	0.854	
At skin closure	0	0.07 ± 0.25	0.155	
Patient satisfaction 1–2–3–4–5 (n, %)	0–0–0–7–23	0–0–0–3–27	0.166	
Umbilical vein PH	7.35 ± 0.04	7.35 ± 0.03	0.751	
Apgar 1 min	9 (9,9)	9 (9,9)	0.647	
Apgar 5 min	10 (10,10)	10 (10,10)	1	

**Note**: Data are presented as mean ± SD or as percentage of group total except maximum sedation level which are number of patients

**Abbreviations**: VAS, visual analogue score

Apgar scores at 1 and 5 min of the neonates and umbilical vein PH were similar between the two groups (Table 3). None of the newborns had an Apgar score < 9.

## Discussion

Our study showed that the ED_50_ of intrathecal dexmedetomidine coadministered with 8 mg or 10 mg plain ropivacaine for cesarean section was 5.9 µg (95% CI, 4.9 to 7.4 µg) or 3.1 µg (95% CI, 0.1 to 4.8 µg), respectively, using probit regression. Hemodynamic stability, side effects, patient satisfaction and neonatal outcomes were similar between the two groups.

The ideal spinal anesthesia for cesarean section should provide adequate surgical conditions throughout the procedure with fewer side effects and earlier recovery of maternal motor functions and should not affect neonatal outcomes. Although a high dose of local anesthetics can achieve adequate intraoperative analgesia and muscle relaxation, it will inevitably lead to a higher incidence of hypotension and motor block [3]. Reducing the spinal dose of local anesthetics can reduce the side effects [3, 4]. However, such a strategy could compromise the adequacy of anesthesia, require supplementary analgesia with possible neonatal consequences, and may necessitate conversion to general anesthesia [3, 4]. Therefore, intrathecal opioids have become a popular choice in the management of spinal anesthesia during cesarean section, which can decrease the dose requirement of local anesthetics, improve the quality of anesthesia, and subsequently aid early ambulation due to faster recovery from motor block [2, 18]. Unfortunately, they have well-known short-term (e.g., somnolence, sedation, pruritus, nausea, vomiting, respiratory depression and urinary retention) and long-term (e.g., dizziness, cognitive impairment, depression, chronic constipation, immunosuppression and structural cardiac changes) adverse effects for patients [19]. Opioid-free anesthesia would yield better postoperative outcomes and is being investigated and advocated as the desirable anesthesia strategy [20].

Several studies have explored intrathecal nonopioids as novel alternatives, such as α_2_-adrenergic receptor agonists [21] and neostigmine [22]. Dexmedetomidine is a highly selective α_2_-adrenergic receptor agonist that has sedative, analgesic, anxiolytic, sympatholytic, amnestic, anti-shivering, and opioid-sparing effects [23]. Studies [7–14] have shown that intrathecal dexmedetomidine can shorten the onset time of local anesthesia, prolong the block duration, and decrease the occurrence of shivering without increasing drug-related side effects; it has been documented to be safe for the mother and neonate at doses ranging from 2.5 to 10 µg. However, the optimal dosage regimen for intrathecal dexmedetomidine has not been determined. The most commonly employed dose of dexmedetomidine was 5 µg [7, 11–14]. Therefore, our study, using the up-down sequential allocation, chose 5 µg as the initial dosage of intrathecal dexmedetomidine to titrate its optimal dosage as an adjuvant to plain ropivacaine for spinal anesthesia during cesarean section.

Although various factors influence the efficacy of nerve block during surgical anesthesia, the local anesthetic dose is the main determinant of its success [24]. We chose 8 mg and 10 mg ropivacaine for the present study, which was based on the findings of previous studies [7, 18, 25, 26]. They found that the ED_50_ of intrathecal plain ropivacaine combined with dexmedetomidine for cesarean section was 9.4 mg [7], 8.1 mg [18] or 6.44 mg [25] when combined with sufentanil. Velickovic et al. [26] showed that ropivacaine (8–12 mg) with opioids for cesarean section may allow ‘walking spinal anesthesia’.

Khaw et al. [27] reported that the ED_50_ and estimated ED_95_ for spinal plain ropivacaine alone for cesarean section were 16.7 and 26.8 mg, respectively. Celleno et al. [28] showed that the ED_50_ of plain ropivacaine for cesarean section using the up-down sequential method was 14.22 mg. The doses of plain ropivacaine used in our study (8 mg and 10 mg) were significantly lower than the optimal doses of ropivacaine alone for cesarean section in the abovementioned studies. It can be inferred that the adjuvant intrathecal dexmedetomidine plays an important role in reducing the dosage of plain ropivacaine.

Earlier mobilization is important for parturients because it enables care for the newborn, reduces the incidence of venous thromboprophylaxis and facilitates early discharge from the hospital. Low-dose local anesthetics in spinal anesthesia permit earlier recovery from motor block and lower maternal side effects [3], but they also result in incomplete motor block, which is considered to be associated with failed spinal anesthesia [27]. In our study, the proportion of patients with incomplete motor block was lower in Group Rop_10_ (3.3%) than in Group Rop_8_ (10.0%); however, the difference between groups was not statistically significant. In contrast, Khaw et al. [27] found that a decreased dose of intrathecal ropivacaine may reduce the degree of motor block accordingly, leading to earlier recovery of motor function. This was likely due to the insufficient sample size of our study. In our study, there was no requirement for conversion to general anesthesia in the patients with incomplete motor block in both groups.

Shivering, associated with spinal anesthesia during cesarean section, is an uncomfortable experience for the parturient; it may lead to increased oxygen consumption, increased peripheral vascular resistance, increased risk of cardiovascular and cerebrovascular diseases, and compromised wound healing [10, 12, 29]. The incidence of shivering related to neuraxial anesthesia was up to 55% according to the literature [29] and may be even higher in cesarean Sects. [8, 12]. In our study, the incidence of shivering was 20% (significantly lower than 55%), without a statistically significant difference between groups. This finding was different from previous studies by He et al. [13] and Bi et al. [8], in which different doses of intrathecal dexmedetomidine differentially reduced the incidence of shivering. The discrepancy may be explained by the fact that the up-down sequential method applied in the present study was not designed to compare the side effect profiles. Mechanistically, dexmedetomidine reduced shivering possibly by stimulating central α_2_-adrenergic receptors and thereby reducing the central thermoregulatory threshold for shivering. It may directly increase the temperature range without affecting thermoregulatory defenses [30].

In our study, no newborn had an Apgar score < 9 in either group, indicating that the dosages of intrathecal dexmedetomidine were safe for neonates. These findings were in line with the results of previous randomized controlled trials [7, 8, 14] and meta-analyses [6].

Our study also has limitations. First, we did not use intrathecal opioids as a control to examine the superiority or inferiority of intrathecal dexmedetomidine vs. opioids. Second, we did not detect the ED_95_ of intrathecal dexmedetomidine, an index more applicable in clinical practice than the ED_50_. In the future, a biased-coin up-down sequential allocation method can be used to determine the ED_95_ of intrathecal dexmedetomidine.

## Conclusions

In conclusion, the ED50 of intrathecal dexmedetomidine as an adjuvant to 8 mg and 10 mg plain ropivacaine in spinal anesthesia during cesarean section was approximately 6 µg and 3 µg, respectively.

### Electronic supplementary material

Below is the link to the electronic supplementary material.

Supplementary Material 1

Supplementary Material 2

Supplementary Material 3

## Data Availability

The datasets used and/or analyzed during this study are available from the corresponding author upon reasonable request.

## Abbreviations

ANOVA: analysis of variance
BP: blood pressure
CI: confidence interval
CS: cesarean section
CSEA: combined spinal-epidural anesthesia
HR: heart rate
ED_50_: median effective dose
SD: standard deviation
VAS: visual analog score
