# Supplementary material for: Intrathecal dexmedetomidine as an adjuvant to plain ropivacaine for spinal anesthesia during cesarean section: a prospective, double-blinded, randomized trial for ED50 determination using an up-down sequential allocation method
Source: BMC Anesthesiol. 2023 Sep 25;23:325. doi: 10.1186/s12871-023-02275-x (PMC10519004; doi:10.1186/s12871-023-02275-x)
Supplement: Supplementary file 1 — Supplementary Material 1 [file 12871_2023_2275_MOESM1_ESM.doc]

**Supplemental table. Score of measurements.**

| **Score** | **Visceral traction response** | **Muscle relaxation** | **Shivering** | **Sedation level** |
| --- | --- | --- | --- | --- |
| 0 |  |  | No perceptible tension of muscles observed | Awake and alert |
| 1 | No discomfort in stomach or perineum; no nausea, vomiting, or intestinal tympanites | No disturbing muscle strain | Slight muscle tonus of masseter muscle | Awake but drowsy |
| 2 | Moderate discomfort in stomach and perineum; no nausea, vomiting, or intestinal tympanites | Disturbing but acceptable  muscle strain | Shivering of proximal muscles | Asleep but arousable |
| 3 | Serious discomfort in stomach; serious discomfort in perineum; obvious intestinal tympanites; nausea and vomiting that require drug treatment | Unacceptable muscle strain | Generalized shivering of the whole body | Not arousable |
