# Supplementary figures and images for: Intrathecal dexmedetomidine as an adjuvant to plain ropivacaine for spinal anesthesia during cesarean section: a prospective, double-blinded, randomized trial for ED50 determination using an up-down sequential allocation method
Source: BMC Anesthesiol. 2023 Sep 25;23:325. doi: 10.1186/s12871-023-02275-x (PMC10519004; doi:10.1186/s12871-023-02275-x)

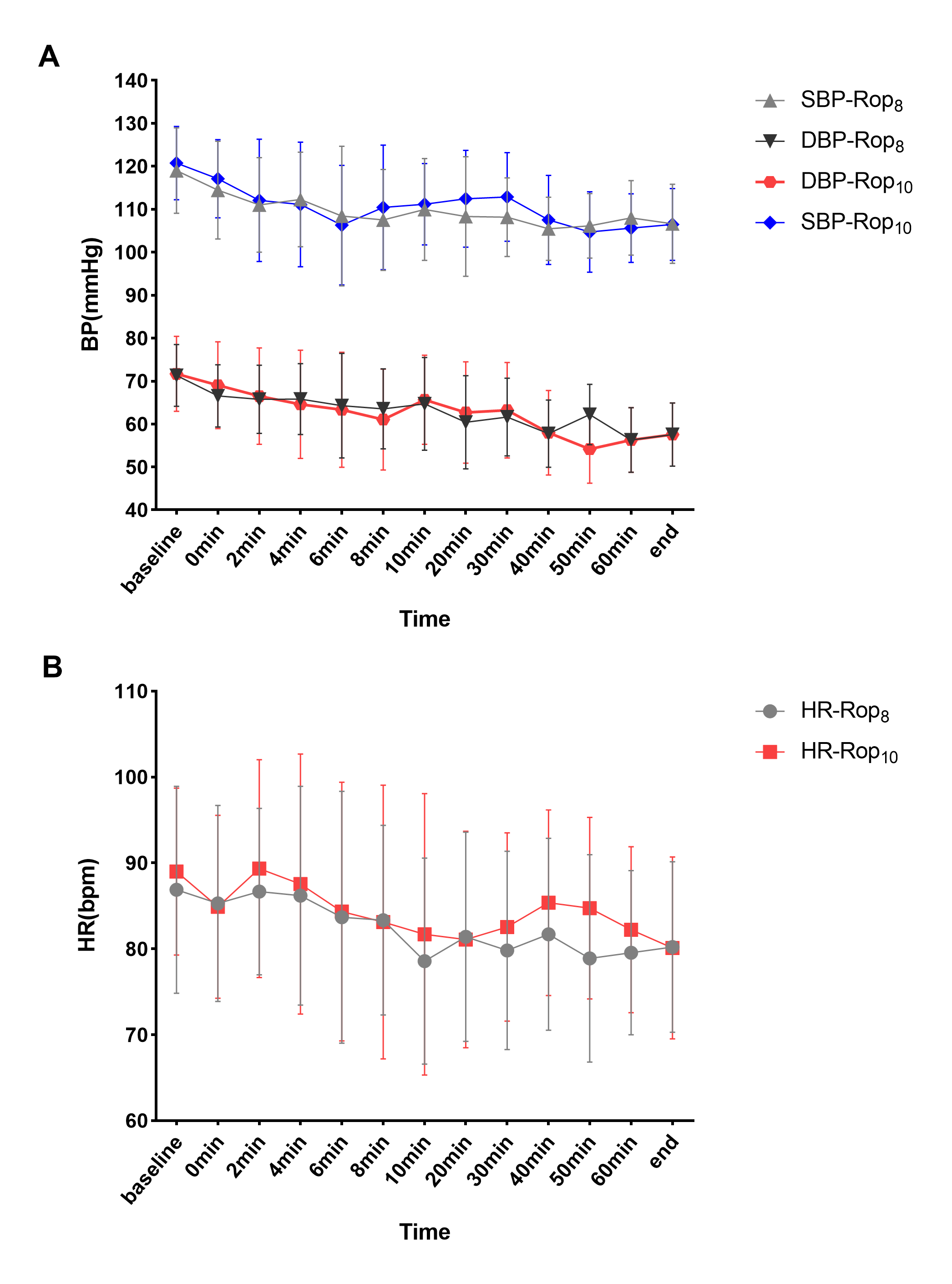

Supplement: Supplementary file 2 — Supplementary Material 2 [file 12871_2023_2275_MOESM2_ESM.png]
