# Supplementary material for: Intrathecal dexmedetomidine as an adjuvant to plain ropivacaine for spinal anesthesia during cesarean section: a prospective, double-blinded, randomized trial for ED50 determination using an up-down sequential allocation method
Source: BMC Anesthesiol. 2023 Sep 25;23:325. doi: 10.1186/s12871-023-02275-x (PMC10519004; doi:10.1186/s12871-023-02275-x)
Supplement: Supplementary file 3 — Supplementary Material 3 [file 12871_2023_2275_MOESM3_ESM.doc]

**Supplemental figure legend**

**Supplemental figure** Hemodynamic parameters. (A) Systolic blood pressure (SBP) and diastolic blood pressure (DBP) between the Group Rop_8_ and the Group Rop_10_. Error bars represent SD. (B) Heart rate (HR) between the Group Rop_8_ and the Group Rop_10_. Error bars represent SD.
